# Physicians’ attitude toward their ethical responsibility regarding air pollution: a qualitative research

**Published:** 2017-09-02

**Authors:** Saeedeh Saeeditehrani, Alireza Parsapour, Saharnaz Nedjat, Maliheh Kadivar, Bagher Larijani

**Affiliations:** 1 *PhD Candidate in Medical Ethics, Medical Ethics and History of Medicine Research Center, Tehran University of Medical Sciences, Tehran, Iran. *; 2 *Assistant Professor, Medical Ethics and History of Medicine Research Center, Tehran University of Medical Sciences, Tehran, Iran. *; 3 *Professor, Department of Epidemiology and Biostatistics, Tehran University of Medical Sciences, Tehran, Iran. *; 4 *Professor, Department of Pediatrics, Division of Neonatology, Children’s Medical Center, Tehran University of Medical Sciences, Tehran, Iran. *; 5 *Professor, Endocrinology and Metabolism Research Center, Endocrinology and Metabolism Clinical Sciences Institute, Tehran University of Medical Sciences, Tehran, Iran; Medical Ethics and History of Medicine Research Center, Tehran University of Medical Sciences, Tehran, Iran.*

**Keywords:** *Air pollution*, *Physician*, *Professional commitment*, *Ethical responsibility*, *Environment*

## Abstract

Air pollution is among the environmental problems that adversely affect people’s health. There is a close relationship between medicine and environment, and as a consequence, there are ethical considerations surrounding the problem of air pollution. The present research aimed to determine physicians’ attitude toward their ethical responsibility regarding air pollution, and their role in reducing it. This was a qualitative research using content analysis, conducted in Tehran University of Medical Sciences. The focus group included 21 physicians with specialties and subspecialties in pediatrics, infectious diseases, pulmonology, gynecology, and midwifery selected through predetermined sampling along with 13 personal in-depth interviews. A number of questions were asked regarding physicians’ ethical responsibility to decrease environmental crises, particularly air pollution.

As a result, 4 themes and 20 subthemes were extracted by assessing the focus group and interviews. These four general themes included the role of a physician as 1) an ordinary person, 2) a special citizen and a role model, 3) a professional person with special personal and social commitments, and 4) an administrator of the healthcare system.

In the present research, physicians acquired a special attitude toward air pollution. The research population mentioned physicians’ impact as role models for the society, as well as their educational, supervisory, informative, promotional, and administrative roles among their most important obligations regarding air pollution. It is recommended to conduct further studies on physicians’ knowledge, attitude and practice regarding their responsibility toward environmental issues in order to investigate this important matter further.

## Introduction

Air pollution is considered a real problem both nationally and internationally (1). Scientific findings indicate several adverse effects of air pollution on human health (2 - 4). The most prevalent side effects of air pollution appertain to the respiratory system (5 - 6). There is also a significant relationship between air pollution and premature birth, infant death and lung capacity reduction (7 - 10). The adverse effects are not limited to the respiratory system; for example, excess lead exposure due to air pollution can cause nervous system complications in children (11), induce an increase in blood pressure among adults (12), and have a negative impact on the cardiovascular system (13).

Air pollution has adverse effects on the reproductive system, pregnancy, fetuses, and infants (14-16). Genetic studies demonstrate the harmful effects of air pollution (17, 18), and there are evidences that it may also result in infertility in men (19). 

Certain symptoms may be direct indicators of exposure to air pollution, such as nausea, chest pain, dyspnea, itchy throat, eye irritation, headache, palpitation, sputum, coughs, , and excessive fatigue (20). Moreover, several studies have demonstrated that air pollution raises the rate of hospitalization significantly (21 - 23) and others have pointed to an increased number of emergency visits when pollution prevails (24).

It is evident that all the costs and side effects ensuing from air pollution appertain to the healthcare system. Thus, the healthcare system and health service providers should have a special ethical sensitivity to environmental problems due to their responsibility to protect people’s physical and mental health; hence, their ethical liability toward the environment is more noticeable, which naturally creates certain roles for them. 

Based on the aforementioned points, physicians’ sensitivity, awareness and attitude with respect to their ethical responsibility play a major part. Studies also demonstrate the influence of physicians’ attitude toward air pollution on changing the public view of the subject (25 - 28). Moreover, due to their status in the society, physicians’ conduct can provide a proper model to contribute to a change of public attitude toward environmental problems, which are basically created by man. 

Air pollution is an example of an environmental problem resulting from activities such as transportation, energy production (transformation), and other energy-related industrial activities, which are the major producers of pollutants (29). Thus, changing the behavior and attitude of the people in a society will be a significant contribution to the reduction of air pollution. 

Physicians’ role in this issue may be different depending on their approach to the subject. Few studies have been conducted on physicians’ moral responsibility regarding air pollution and its status in physicians’ personal and professional priorities, or the attitude of this significant social class toward their role in reducing environmental crises. Consequently, it is of utmost importance to investigate the above-mentioned issues in order to provide the opportunity for interventions (for instance through altering or consolidating physicians’ influential attitude), and create the grounds for conducting further studies to discover the obstacles and deterrents. Therefore, this study endeavors to elicit the ideas of physicians and experts as professionals who are notably involved in the issue, and solicit their opinions on their own ethical role and responsibility regarding the reduction of air pollution. 

## Method

This study was conducted between March 2014 and February 2016 in Tehran University of Medical Sciences. It investigated physicians’ attitude toward their role in reducing air pollution and presented the personal and public solutions offered by this social group. In order to discover the profound and diverse aspects of the issue, it seemed necessary to apply a qualitative methodology, and therefore the methodology used in this research was “content analysis” (30, 31). 

The data collection method consisted of semi-structured interviews and focus group interviews. First, a group discussion took place with the presence of 21 physicians, including 7 pediatricians, 2 neonatologists, 2 fellowship specialists in high risk pregnancy and perinatology, 3 medical ethics experts, and 6 asthma specialists and allergists. The session started with the statement of the problem by the researcher followed by a real medical case; then, several questions were asked and the subject matters were discussed. After implementing and evaluating the session, in-depth interviews were conducted in order to complete the data, as the researcher inferred that more comprehensive results could be achieved by spending more time and conducting personal interviews. Subjects were selected by purposeful sampling with maximum variation and grouped based on gender, age, specialty and rate of clinical experience. These four indicators were chosen because they were easy to identify and evaluate, and not only affected the objective of the study, but also had sufficient variation among the participants. After the interviews began, more subjects were added through theoretical sampling. Since the authors aimed at studying different opinions, the samples were selected out of four groups: pediatricians, pulmonologists, medical ethics experts, and internists. Thus, based on the reference review mentioned in the introduction, those specialists who were more involved in air pollution and the resulting side effects and costs were selected as samples. Prior to the interview, a text containing the guideline to questions and a summary of research objectives were sent to the participants via email. All interviews were recorded with participants’ consent and permission from the ethics committee of Tehran University of Medical Sciences (No. 1686-1395). Each interview began with 5 open questions. The questions were designed in a general and unbiased way in order to encourage the participants mentioning any item they considered valuable in this field (table 1); then the follow-up question were asked based on the information provided by the participants to clarify the construct. Questions for further interviews were prepared based on extracted classifications. Sampling continued until saturation, i.e. when the researcher felt certain that no new data could be added anymore, and continued up to 13 samples (3 internists, 2 pulmonologists, 1 dermatologist, 4 pediatricians, and 3 medical ethics experts). The last two interviews contained similar information, and the researcher was satisfied with adequacy of interviews.

**Table 1:** General questions

Concepts of Questions
The relationship between health and environment
Physicians’ role in air pollution and the related side effects, and the obstacles to the realization of their role in air pollution reduction
The role of policymakers and administrators in reducing air pollution

To analyze the data, the present research used “the Corbin & Strauss constant comparative method” consisting of three stages of open coding, axial coding, and selective coding (32, 33). During the open coding stage, the texts were first implemented and then reviewed and reread multiple times. Researchers examined the data and extracted the major points and constructs, then gave a special code to each sentence. Lincoln and Guba’s evaluative criteria method was applied to validate the data (34) and the validity of all eight criteria of this qualitative research was confirmed. 

The researcher’s long-term involvement in the research atmosphere and constant observations were considered. The researcher spent fourteen months collecting evidence from various resources, while external research was controlled by a third party arbitration, that is, the medical ethics expert who was not involved in the research. Triangulation and negative case analysis were performed by the researcher, and the discussions were presented using thick description. As a final stage, member check was applied to increase validation.

## Results

*Participants and procedure*

Twenty-one physicians (55% male and 45% female) participated in focus groups and 13 in interviews. Their average age was 40, and they had an average of 18 years (±10) of clinical experience. The focus group sessions lasted 100 minutes, and the average duration of the interviews was 30 - 45 minutes. Researchers coded each transcript independently, which resulted in extraction of 887 codes from the focus groups, and 105 codes from the interviews. A thematic analysis was performed after each interview and focus group session.

Interpretation of the coded texts enabled classification of the codes and establishment of the relationships between the various codes or categories. The codes were compared and discussed until agreement was reached. After classification, various categories were identified on the basis of descriptive hypotheses, and 4 themes and 20 subthemes were extracted overall. 

**Table 2:** Evaluation of physicians’ role in mitigating air pollution and environmental problems

**Themes**	**Subthemes**	**Codes**
**Physicians’ role as ** **ordinary citizens**	Observing environmental issues as ordinary citizens	Physicians are ordinary citizens, and are effective and influential in their role as members of the society. Since air pollution and the environment are public concerns, physicians ought to observe these issues as other individuals, for instance use their personal cars less frequently, use public transportation, avoid smoking, contribute to tree planting, observe regulations such as vehicle inspection, and so on.
**Physicians’ role as ** **special citizens ** **and role models**	Physicians must have an appropriate conduct toward the environment	Physicians have a distinctive position as special citizens and role models. Physicians are role models in the society and can influence the perspective of the general public. Therefore, they have greater individual and social obligations, and are more influential in culture making. People believe in physicians. As individuals, physicians have two roles: a) improving behavior b) teaching.
	They can teach others directly	If physicians are concerned with the environment, they can make contributions through activities such as making posters conveying environmental messages, separating dry and wet
	They can teach others implicitly (by providing role models)	waste in the clinic, and avoiding improper methods of garbage disposal. Such acts have a promoting and educational effect on patients. Physicians’ professional conduct is especially significant; if physicians separate their wastes or have a healthy conduct, they can influence their patients’ and others’ outlook. This is also a form of education for patients and a way of culture building for the society, since such physicians not only behave properly and professionally, but also educate the society. As an example, physicians’ use of public transportation has a tremendous educational effect on other members of the society.
**Physicians’ role as ** **professionals with ** **special ethical ** **liabilities and ** **sensitivities, both ** **personal and ** **social**	Writing papers on air pollution and its adverse effects on human health	Physicians are influential because they can publish health- related articles. For example, articles showing accurate statistics about the dangers of air pollution in various organizations. They can also attract the attention of policy makers and managers to this important issue.
	Presenting real statistics and reports for informing the healthcare system, the society, and the public	Reliable, scientific and updated articles, statistics and reports can attract the attention of associations and scientific centers now more than ever.
	Conducting relevant research projects about air pollution and its harmful effects on health Informing and raising awareness in three levels: public associations, policy- making, and the society	Physicians and other health care providers must understand the need for research in this field Reliable scientific articles with real statistics can cause the public to look into the issue. This raises awareness both in the scientific community and across the whole society. It can also have a significant impact on the general outlook and even direct it.
	Monitoring air pollution in a more systematic way, for instance on a monthly or daily basis	Provision of monthly and annual detailed statistics to the Ministry of Health and other related organizations can be very effective in making changes in the present state of affairs.
		There are few scientific papers and detailed statistics on air pollution and its harms to the environment. It is important that physicians be concerned about this issue.
	Showing sensitivity to prevalent environmental crises; in general, professional physicians should have a comprehensive insight and show sensitivity to environmental crises	Monitoring environmental problems and responding to them is essential. It is important that physicians be concerned about the environment, especially air pollution. Factories that are built around large cities all contribute to pollution. Such problems arise when people care only about economic interests and plan without first conducting the necessary environmental studies.
	Observing some points by physicians and the healthcare community (in the clinic, hospital, or laboratory) which decrease air pollution. All physicians should evaluate the circumstances in their clinic or hospital in order to reduce air pollution in their professional domain	Likewise, employment and development are important, but all should be in line with the environment. Deforestation, irresponsible urban development, car manufacture, production of non-standard cars and fuels, and destruction of the environment are very important issues, and the medical community should also show sensitivity towards them.
	Social commitments based on the attention of health organizations to the importance of the subject, and designing the necessary models for environment-protecting clinics, laboratories, or hospitals	Physicians and health care providers should start from themselves. Every physician should evaluate issues in their workplace and address problems that can be resolved by themselves or the hospital. Gynecologists stated that the incubator machine produces a type of gas harmful to children, and the older the incubator, the higher the produced gas rate will be, an item mostly neglected by physicians. Moreover, physicians have some frequent unnecessary visitors, an issue that can be taken care of by telemedicine, or phone and internet communication. Repetitive unnecessary visits to physicians can be reduced through tele-medicine via phone or the internet.
		Green hospitals and developing organizational guides for the staff to promote environmental behavior in the workplace is very effective.
		Prevention is also another responsibility of physicians, and solving environmental problems is related to this issue.
	Professional commitment to treatment and follow-up of the patients	Treating patients is a major duty, but the harms and damages that air pollution induces on health should also be prevented. Drinking milk, for instance, can prevent the adverse effects due to the calcium in milk, or vitamin C intake is useful because of its antioxidant feature. Providing information on such subjects by physicians is very important for patients and the society alike.
		Physicians can offer solutions for vulnerable groups to help them become less affected by air pollution, for instance by introducing a type of nutrition beneficial to children, or providing masks to pregnant women.
	Professional commitment to paying special attention to the health of vulnerable groups	As another vulnerable group, women should not to be exposed to air pollution, and must be encouraged to drink milk to protect them from the adverse effects. Likewise, the elderly should stay at home or avoid exercising in polluted air.
**Physicians’ role as ** **administrators of ** **the healthcare ** **system, and their ** **environmental ** **responsibilities**	Creating codes and directives on green hospitals, laboratories and faculties	In this regard, physicians and hospitals must take serious actions. Green hospitals can contribute to environmental protection. Having Green hospitals and developing organizational guide the staff to have effective environmental behavior in the hospital. This strategy is very effective.
	Consulting with experts and patients and attracting the beneficiaries’ participation	Physicians should address the psychological and social aspects of the issue, which often necessitates consultation with colleagues and talking with patients and their relatives. It is also important to reach an agreement and get help from others if necessary and obtain the participation of all stakeholders.
	Teaching environment protecting behavior by university authorities Supporting environment-related research (on air pollution): if healthcare authorities support research projects, physicians and experts will pay heed to such activities more carefully	Education is very important for medical students, patients and other people, and physicians and health care managers can contribute. Culturalization begins with universities and academic environments. Therefore, professors and university presidents can educate the students in environmental issues. The faculty and the department must support air pollution research and ask physicians to present related articles and reports. Follow-ups by the authorities help and create an urge for physicians and experts in the field. Healthcare providers and health policy makers are responsible for making the health industry green. They should implement clean and green policies in hospitals, health centers, laboratories, and university departments. For example, green hospitals have specific codes. In general, health system actions on the policy- making level must be environmentally friendly. Health authorities should constantly think and function in environmental terms.
	A serious request from experts and physicians to present reports and scientific papers in the field	
		Physicians should review research plans that may potentially harm the environment.
	Reviewing all research plans in terms of environmental threats (specialty committees)	
	Healthcare system administrators should expect answers and explanations regarding environmental crises from authorities	Physicians may submit reasonable requests to related centers, such as public transportation or environment organizations, and urge activities to reduce air pollution. They may even go as far as observing environmental problems and showing proper and timely reaction to environmental degradation (factory building, deforestation, drying the lakes and rivers). Furthermore, they can acquire a comprehensive insight and study the effects and outcomes of environmental degradation.

*First theme: Physicians’ role as ordinary citizens:*

One subtheme was extracted in this regard, which was observing environmental points as ordinary citizens. More explanations have been provided in table 2.

*Second theme: Physicians’ role as special citizens and role models:*

There are three main subthemes in this item: a) a physician must have an appropriate conduct toward the environment; b) he/she can directly teach others; c) he/she can implicitly teach others (Table 2). 

*Third theme: Physicians’ role as professionals with special ethical liabilities and sensitivities, both personal and social:*

- Physicians should write relevant papers regarding air pollution and its adverse effects on human health. This issue was considered particularly valuable and was pointed out in most interviews.
- Physicians can present real statistics and reports. This is very useful for informing the healthcare system, society, and public, as the statistics given by this social class will be scientifically accurate, and appropriate solutions can be based on them.
- Conducting relevant research projects on air pollution and its harmful effects on health will be extremely helpful.
- Informing should take place on three levels: public associations, policy-making, and the society. Physicians have certain social commitments and should be sensitive to affairs that jeopardize the public health. Informing can be useful at all levels, either at the social or policy-making level. In addition to statistics and papers mentioned earlier, informing can be done in the form of interviews and technical meetings with the media, social networks, and so on.
- Monitoring air pollution in a more systematic way, for instance on a monthly or daily basis, is especially important. Those who are engaged in environmental activities, particularly physicians, are expected to monitor environmental issues more systematically since they are much more involved in air pollution costs and harms thanks to the facilities and possibilities available for them.
- Physicians need to be sensitive to prevalent environmental crises; in general, professional physicians should have a comprehensive insight into such issues, because the majority of environmental crises have a direct or indirect impact on human health.
- In clinics, hospitals and laboratories, physicians and members of the healthcare community must observe certain details regarding environmental protection. More specifically, physicians should evaluate measures to be taken in the clinic or hospital in order to reduce air pollution in their professional domain (Table 2).
- Physicians have social environmental commitments based on the attention of health organizations to the importance of the subject, and they can help design the necessary models for environment-protecting clinics, laboratories, or hospitals (Table 2).
- Physicians have the professional commitment to treat and follow up their patients (Table 2).
- Another professional commitment of physicians concerns the provision of special care to the health of vulnerable groups (Table 2).

*Fourth theme: Physicians’ role as administrators of the healthcare system, and their environmental responsibilities:*

- Physicians are required to create codes and directives on green hospitals, laboratories and faculties. There is great need for such establishments, and writing organizational guides on the staff’s conduct in relation to environmental protection is also useful.
- Physicians can consult experts and patients and attract the beneficiaries’ participation in environmental affairs.‌ They may consider the mental and social aspects and problems and consult their colleagues, patients and their families to reach an agreement and resolve the issues; furthermore, they can ask other people for help if necessary, and try to attract the beneficiaries’ help.
- University authorities should also teach and promote environment protecting behavior. Instructing patients, medical students and other members of the society is very important, and physicians and healthcare system administrators can participate in the process. Culturalization initiates from universities and academic institutions; therefore, professors and heads of universities can educate their students on environment-related issues and principles.
- If healthcare system authorities support environment-related research projects (in this case on air pollution), physicians and experts will pay heed to such activities more often. Participants’ examples are presented in Table 2.
- There is serious request from experts and physicians to present reports and scientific papers on the field.
- Specialty committees should review all research plans in terms of potential threats to the environment. For instance, ethical committees should consider the constructive points in research projects that contribute to protect the environment.
- Healthcare system administrators should expect answers and explanations regarding environmental crises from government authorities. Moreover, they may request environment-related action from all centers involved. Participants’ examples are presented in Table 2.

## Discussion

As health service providers, physicians face diseases and complications that result from environmental crises such as air pollution every day. As citizens in general and as professionals in particular, they are expected to assume responsibility for environmental crises. The present research was conducted to examine how they can participate in the process of reducing air pollution. This study showed that physicians believe they can be effective in this field on four levels:

**Level 1: ** In their role as ordinary citizens, physicians resemble other citizens in that they are clearly expected to perform their duty to protect the environment. Other studies have mentioned this role as well, for instance, Lorenzoni et al. stated that those employed in the healthcare system are ethically responsible for the environment as individuals, both in their personal and social life (35). Islamic ethics also frequently recommends the protection and reconstruction of the environment. Influential Islamic doctrine emphasizes that the nature belongs to God, so human beings are always present in the divine land, even if they are not aware of it. According to Islamic teachings, environmental crises are the outcome of people’s negligence in their responsibilities toward the nature, and their wrong belief that the environment is separate from the divine land. Thus, Islamic thoughts always urge citizens to protect and preserve the environment (36 - 38).

**Level 2:** A physician is also a special citizen and a role model whose conduct is emulated and followed by the society. A physician’s conduct can be educational for the patients and others, both directly and indirectly.

**Level 3: **The next level mentioned in this research was a physician’s role as a professional with special personal and social commitments. This role is of utmost importance, as WHO also defined the responsibility of the healthcare system and hospitals beyond mere treatment and prevention of diseases in 2009, stating that hospitals should align their policies with environmental and climate changes due to the effect of such factors on public health (39). The physicians in the present research also mentioned supplementary endeavors that show significant professional commitment to the subject. Instances included writing papers, presenting reports, and informing the public, all of which may take place on three levels: in scientific centers, policy-making, and the society. Global studies also indicate that health service providers should be ethically sensitive to environmental problems because of their immediate and direct impact on human health. For instance, climate change and the related issues have a serious effect on people’s well-being, so the healthcare system is professionally expected to consider them (40). The common point between the present research and other studies was the importance of healthcare providers’ direct and indirect role in environmental problems that affect not only the general health but also the environment (41). The next point achieved in the research was showing sensitivity to these problems and taking a comprehensive view on the matter. This topic has been discussed and agreed on in some studies, albeit in a different light; for example, a qualitative study conducted in 2015 on 18 nurses demonstrated that the nurses’ view of their duties and responsibilities is mostly confined to their job-related tasks. They turned out to prioritize hygienic issues such as infections, and did not have a comprehensive insight on climate change or environmental problems, or at least did not prioritize them even when aware of their importance (42). Meanwhile, a 2005 study conducted in Paris insisted on the necessity of healthcare providers’ comprehensive concern for environment, since environmental issues can play a major part in every individual’s health in general (43). A 2015 study in the UK showed that those in the medical profession display their individual and organizational training and leadership roles in issues such as release of penicillin, tobacco control and enforcement of vehicle safety belt laws. Relying on such instances, the present study refers to physicians’ important role in resolving social problems and urges a similar role in environmental issues (44).

Thus, a medical treatment team with heightened sensitivity to the environment may not only direct their professional conduct toward environmental protection, but also will look at environmental problems in a professional light and present their views on public health to policymakers. In fact, therapists are one of the best groups to observe and study environmental problems and provide policymakers with appropriate data.

**Level 4: ** Physicians also have the role of caretakers of the health and education system. This role can be categorized into two groups: reforming educational policies and directing them toward the attitudes resulting in proper conduct, and appropriate policymaking to protect the environment as much as possible. 

This study reviewed a new concept of environmental responsibility within the framework of physicians’ social responsibility, presenting it as physicians’ role in environmental issues. These roles are summarized as follows:

*The first role*

Teaching environmental ethics to students by university authorities was a pivotal point mentioned in this case. As centers of education and culturalization, faculties are highly important. Informing the students will contribute greatly to changing their attitude and behavior, and consequently those of the society. A 2011 study on senior medical students of medicine examined their attitude toward climate change and demonstrated that their opinions were changeable and resulted from the general attitude of the healthcare system toward environmental problems (45). Thus, the attitude and conduct of authorities will significantly affect the students’ behavior. 

*The second role*

Three domains can be mentioned in this respect: presenting proper executive policies, supporting research projects, and review and supervision. One of the subjects discussed here was regulation of codes and directives for green hospitals, laboratories and faculties. This is extremely useful and fundamental, as many renowned medical faculties all over the world have regulated related ethical codes and directives (46, 47). Committees and unions throughout the world have discussed this important issue in their ethical codes. For instance, the International Council of Nurses published nurses’ ethical codes in 2012, taking into consideration the environmental issues and observations (48). Unfortunately, there are no such ethical codes in Iran, and aside from the ethical code of research on animals (49) and medical research codes (only in one paragraph) (50), there is no clause among the thirteen written codes which specifically address environmental issues.

Another item related to this role is the support of research projects. The subthemes mentioned in the last topic as physicians’ professional duties depend on the support of healthcare system administrators in order to be realized. Thus, writing papers, presenting statistics, preparing scientific and valid reports, and conducting relevant research on the subject require the support of healthcare system authorities. Moreover, in order to establish green hospitals and make proper use of telemedicine, the essential substructures should be provided by healthcare system administrators, and research ethical committees should be bound to study and assess the rate of pollution from an ethical viewpoint. Supervising this vital item is one of the duties of the heads of faculties. 

*The third role*

Healthcare system administrators should ask the authorities for explanations regarding environmental crises, because although problems such as air pollution are environmental in nature, the healthcare system will be responsible for the resulting harms and damages. Therefore, ethical sensitivity and concern about such problems are essential for healthcare system authorities. 

To summarize, environmental issues should be taken into consideration in the light of medical ethics to determine the ethical duties of the medical society.

## Limitations of the study

Since the topic was rather new, some of the experts did not have an adequate mental background in the subject and were therefore unable to cooperate in their full potential. In order to remove this obstacle, we provided them with the question guide (42). Furthermore, in spite of the attempt to reach data saturation in the studied specialties, some of them may not have been prioritized in the interviews; however, almost all of the relevant specialties were considered according to the literature review.

## Conclusion

Ideas on medical professionalism at all three levels (interpersonal, intrapersonal and public) and physician’s social responsibilities are frequently overlapping. Both require the physician to be accountable to the communities and the society in which they serve. Therefore, the physician should be increasingly prepared to respond to the growing environmental challenges and their social consequences.

The present research shed a light on some aspects of this mutual relationship, suggesting that policymakers consider these aspects when establishing laws, supervisory mechanisms, and educational and research policies to create a positive attitude and control physicians’ conduct regarding environmental issues. Since there is not much positive attitude in the field of environment, one of the priorities of the healthcare system can be to establish an appropriate attitude through different means, for instance by way of education.
